# Risk of travel-related cases of Zika virus infection is predicted by transmission intensity in outbreak-affected countries

**DOI:** 10.1186/s13071-017-1977-z

**Published:** 2017-01-25

**Authors:** Nicholas H. Ogden, Aamir Fazil, David Safronetz, Michael A. Drebot, Justine Wallace, Erin E. Rees, Kristina Decock, Victoria Ng

**Affiliations:** 1 National Microbiology Laboratory, Public Health Agency of Canada, rue Sicotte, Saint-Hyacinthe, Québec Canada; 2 National Microbiology Laboratory, Public Health Agency of Canada, Research Lane, Guelph, ON Canada; 3 National Microbiology Laboratory, Public Health Agency of Canada, Arlington Rd., Winnipeg, MB Canada

**Keywords:** Zika virus, Travellers, Risk, Basic reproduction number

## Abstract

**Background:**

Zika virus (ZIKV) infection is emerging globally, currently causing outbreaks in the Caribbean, and Central and South America, and putting travellers to affected countries at risk. Model-based estimates for the basic reproduction number (*R*_*0*_) of ZIKV in affected Caribbean and Central and South American countries, obtained from 2015 to 2016 human case surveillance data, were compared by logistic regression and Receiver-Operating Characteristic (ROC), with the prevalence of ZIKV-positive test results in Canadians who travelled to them.

**Results:**

Estimates of *R*_*0*_ for each country were a good predictor of the ZIKV test result (ROC area under the curve = 0.83) and the odds of testing positive was 11-fold greater for travellers visiting countries with estimated *R*_*0*_ ≥ 2.76, compared to those visiting countries with *R*_*0*_ < 2.76.

**Conclusions:**

Risk to travellers varies widely amongst countries affected by ZIKV outbreaks. Estimates of *R*_*0*_ from surveillance data can assist in assessing levels of risk for travellers and may help improve travel advice. They may also allow better prediction of spread of ZIKV from affected countries by travellers.

**Electronic supplementary material:**

The online version of this article (doi:10.1186/s13071-017-1977-z) contains supplementary material, which is available to authorized users.

## Background

Zika virus (ZIKV) infection is a globally emerging infectious disease that is, at the time of writing, particularly affecting countries in the Caribbean, and Central and South America [1]. Outcomes of ZIKV infection are mild in most cases but in recent outbreaks more severe outcomes, including Guillain-Barré syndrome (GBS) and foetal central nervous system developmental abnormalities (particularly microcephaly), are now recognised [2]. It is thought that ZIKV is primarily transmitted in tropical and subtropical regions by the mosquitoes *Aedes aegypti* and *Ae. albopictus* [3, 4], but sexual transmission also occurs in humans [5–8]. However, to date epidemic/endemic transmission of ZIKV has been limited to tropical and subtropical regions (according to data in [4]). This suggests that sexual transmission, or temperate zone mosquito species, are not capable of maintaining ongoing transmission on their own in the absence of tropical/subtropical *Aedes* spp. vectors [4, 9] and/or a climate warm enough for ZIKV transmission by these species [4, 10].

To manage risks for residents of ZIKV-free countries there are two key questions: (i) How does risk for travelling citizens vary from one affected country to another? This is a function of environmental factors (e.g. climate, habitat, altitude) and socioeconomic factors (e.g. population density, the built environment and the capacity for countries to control infection) that determine the location-specific efficiency of virus transmission cycles and thus the abundance of human-biting infective vectors [11–13]. (ii) From which affected countries are returning travellers most likely to come with ZIKV viraemia and possibly capable of seeding endemic transmission? This also depends on the abundance of human-biting infective vectors, but also on the total numbers of travellers and the proportions that use air travel, the speed of which allows infected individuals to travel long distances while still viraemic [14]. The proportions of travellers infected may simply correlate with the force of infection in affected countries. However, many travellers may go to holiday resorts or other locations where the risk of infection is dramatically different from that of the resident populations.

The intrinsic location-specific efficiency of mosquito-borne virus transmission cycles determines the level of risk of Zika virus infection to which travellers are exposed, and during the initial phase of an outbreak this risk of infection is positively associated with the basic reproduction number (*R*_*0*_) of Zika virus in the affected country. *R*_*0*_ is the number of new cases of disease in humans produced by one case in a naïve population under the conditions of the particular location under study, and is the gold-standard metric of the capacity of an infectious agent to propagate [15].

In this study we assess the relationship between the proportions of Canadian travellers that test positive for ZIKV after travelling to countries where ZIKV is or has recently been epidemic, and estimates for *R*_*0*_ in those countries obtained from human case surveillance data. We find evidence that these are related, suggesting that estimation of transmission efficiency in affected countries may be used to nuance assessments of risk for travellers, and allow more precise estimates of the rates of introduction and spread of ZIKV by ZIKV-viraemic travellers.

## Methods

### Zika surveillance data

Data for 39 countries and territories of the Caribbean, and Central and South America with confirmed autochthonous transmission of ZIKV were obtained from the Pan American Health Organization [16]. Data extracted on June 16, 2016 contained 377,525 ZIKV cases (364,030 suspected and 13,495 confirmed cases) reported between August 15, 2015 and June 11, 2016. Case counts were reported by epidemiological week with a date corresponding to the first day of that week. PAHO provides recommendations for case definitions of confirmed and suspected cases [17]; however, case definitions vary amongst countries and these are detailed in Additional file 1. For this study, confirmed and suspected cases were combined for each country due to the varying case definitions and degree to which confirmed and suspected cases are reported.

### Estimation of the basic reproduction number (*R*_*0*_)

The Incidence Decay and Exponential Adjustment (IDEA) model was used to estimate *R*_*0*_ for each ZIKV affected country. The IDEA model is a one equation, mathematical model that can be used to describe epidemic dynamics when only basic epidemiological information is available [18]. The basis of the IDEA model is that in the absence of intervention or population immunity, the number of cases will grow exponentially with each serial interval (*t*; the time it takes for infection in one person to give rise to infection in another person *via* transmission by a mosquito) by a factor that is *R*_*0*_. In reality, epidemics decay within a timeframe due to processes such as increases in population immunity and/or control efforts of one form or another. The IDEA model accounts for this decay by estimating a “control factor”, *d* which represents all dynamic processes that slow epidemic growth (e.g. public health interventions, declining vector abundance, and increasing herd immunity). The IDEA model form is:

$$ {I}_t={\left(\left({R}_0/{\left(1+d\right)}^t\right.\right)}^t $$

where *I*_*t*_ is the number of new cases in a serial interval *t*, *R*_*0*_ is the basic reproduction number, and *d* is the control factor [18].

Using this equation, estimates for *R*_*0*_ and *d* were calculated from the changes in case counts observed over successive serial intervals in the ZIKV surveillance data during the epidemic in each affected country. The optimization tool, Solver, in Excel 2010 (Microsoft Corporation, Redmond, WA, US) was used for these estimations. The sum of least squares approach was used to estimate the best fit for *R*_*0*_ and *d* by minimizing the sum of the squared deviations between the observed case counts and the predicted case counts estimated by the model.

For these calculations, time zero of the epidemic in each country was assumed to be the date of the first reported confirmed or suspected ZIKV case. However, in 19 countries multiple cases were reported in the first week of reporting. For these countries a burn-in period was employed, which was the number of weeks prior to the first week of reporting that the first case likely occurred. This number of weeks was simply calculated as the number of times the case counts in the first week of reporting could be divided by two before a case count of one was reached.

The IDEA model requires data input in the time scale of the serial interval (*t*), so PAHO case data were transformed from weekly case counts to corresponding serial intervals. We selected a serial interval of 16 days based on best estimates of 15 to 16 days with a possible range of 10 to 23 days [19]. To rescale weekly PAHO data into a 16-day serial interval, the PAHO data for each country was linearly interpolated between weeks to obtain estimates of daily case counts using Stata/IC for Windows version 14.1 (College Station, TX, US). Then weekly case count data were matched to each 16-day serial interval (or other serial intervals for sensitivity analysis).

### Testing of travellers

All ZIKV test data used in this study were obtained by testing at the National Microbiology Laboratory (NML), Public Health Agency of Canada. The diagnostic algorithms for ZIKV testing in Canadians followed guidelines developed by the Committee to Advise on Tropical Medicine and Travel [20] and mostly agree with recommendations of the United States Centers for Disease Control and Prevention (CDC, [21]) and the World Health Organization [22]. Testing for ZIKV was recommend for: (i) travellers returning to Canada from areas with known or highly suspected ZIKV transmission and who experienced symptoms consistent with ZIKV infection; (ii) asymptomatic pregnant women with travel history to affected regions; and (iii) those having recent sexual contact with a partner who was confirmed to be infected with ZIKV. As the latter type of exposure would not be associated with travel, data on these tests were not included in this study.

ZIKV testing was conducted using diagnostic assays developed and validated by CDC [23]. Acute sera collected within 10–14 days post-onset from symptomatic individuals were first tested for the presence of virus by RT-PCR [23] with samples considered positive if both the gene targets in the PCR were positive. If negative, sera were tested by an IgM-antigen capture enzyme-linked immunosorbent assay (MAC-ELISA) and confirmed by plaque reduction neutralization test (PRNT) [23]. The PRNT assay was conducted in parallel with a dengue virus PRNT assay and only samples that yielded a 4-fold or higher titer for ZIKV were considered positive.

The dataset used in this study comprised all ZIKV tests performed at NML on individuals returning to Canada with a travel history to a ZIKV affected country or territory up to June 21, 2016. The de-nominated dataset included a case ID, the date the test sample was received by NML and information on country/territory or countries/territories to which the case had recently travelled (although precise dates of travel were not available). A total of 5157 unique test samples (of which 111 were positive) with a history of travel were received between May 27, 2015 and June 17, 2016. However after eliminating samples that did not precisely identify countries or territories of travel, travel to potentially Zika-affected countries or territories outside of the Americas and those from travellers who visited multiple Zika-affected countries or territories there were 4533 test results available for the analysis.

### Statistical analysis

The shape of the relationship between the likelihood of a positive ZIKV test result and *R*_*0*_ values was investigated by lowess smoothed estimation of the log of the odds ratio for testing positive [24], to see if polynomial forms or categorisation of the explanatory variable were needed for logistic regression analysis [25]. The relationship approximated to a step function so for analysis the data were categorised into two groups with high and low estimated *R*_*0*_ values. The performance of *R*_*0*_ values as a predictor of the test result was assessed using the receiver operating characteristic (ROC) area under the curve (AUC), and in so doing, values for sensitivity and specificity of different cut-off levels of *R*_*0*_ for predicting the test result were generated. ROC analysis provided a basis for selection of a *R*_*0*_ cut-off value to categorise the *R*_*0*_ values into one low (value = 0) and one high (value = 1) group for logistic regression analysis. The *R*_*0*_ cut-off value chosen was that which gave the highest Youden index (= Specificity/100 + Sensitivity/100 – 1, i.e. giving equal weight to specificity and sensitivity [26]). To quantify the difference in the likelihood of travellers testing positive after visiting countries with high versus low *R*_*0*_ values, logistic regression was run using ZIKV test result as the outcome and the dichotomised *R*_*0*_ value groups as the explanatory variable. To account for unmeasured sources of variation amongst countries visited, an ID number for the country visited by the traveller was included as a random effect. Analyses were undertaken using Stata/SE for Windows version 14.1 (College Station, TX, US) with the level of significance set at *P* < 0.05. Data used in the analyses are available in Additional file 2.

### Sensitivity analysis

The only variable in the IDEA model that can affect estimation of *R*_*0*_, and which can be varied by the user, is the serial interval (*t*). We used a serial interval of 16 days for the main analysis, however this figure is estimated from limited data [19] and will likely vary with different environmental conditions including different seasons, mosquito biting rates and extrinsic incubation period lengths. Therefore a sensitivity analysis was conducted to assess how variations in the value of *t* may impact the results of the study.

To do this, additional *R*_*0*_ estimates for each ZIVK-affected country were estimated using the IDEA model for one lower value for *t* (10 days) and one higher value (23 days). The performance of the new *R*_*0*_ values as predictors of the test result was assessed as above using the ROC AUC. The significance of differences in the AUC values using *R*_*0*_ values obtained using the three different values for *t* were assessed using the roccomp procedure for testing the equality of two or more ROC areas [27] in Stata/SE for Windows version 14.1. A cut-off level for each of the two new *R*_*0*_ values, which gave the highest Youden index values, was chosen to dichotomise the *R*_*0*_ values into low high groups as described above. Logistic regression models (one for each set of *R*_*0*_ values obtained with 10 and 23 day values for *t*) were then run using ZIKV test result as the outcome and the dichotomised *R*_*0*_ groups as explanatory variable with the ID number for the country visited by the traveller included as a random effect. All analyses were completed using Stata/SE for Windows version 14.1 with the level of significance set at *P* < 0.05.

## Results

### Zika surveillance and test result data

**Fig. 1:**
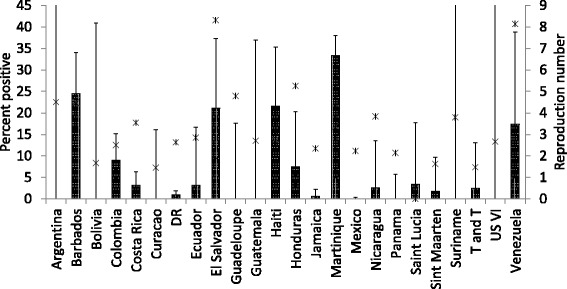
The prevalence (as a %) of ZIKV-positive test results (with exact 95% confidence intervals) in travellers visiting the ZIKV-affected countries on the x-axis. *Stars* indicate the basic reproduction number (*R*_*0*_) estimates for each country. *Abbreviations*: DR, Dominican Republic; T and T, Trinidad and Tobago; US VI, US Virgin Islands

Of the 4533 Canadian traveller test results with information on travel destination to a single country, 104 tested positive and these travellers had visited one of 46 countries or territories in Central and South America. Of these countries or territories there were 39 for which ZIKV surveillance data were available. However data from Belize, Cuba and Grenada were not used because of very low case counts at the time of extraction. Data from Brazil were also not used as they suggested that surveillance began close to the peak of the epidemic and back-interpolation to identify the approximate date of the index case was considered unreliable. Furthermore, surveillance for non-microcephaly cases ceased in early 2016 [28]. Data from Aruba, Paraguay and Peru were not used because the ZIKV case data were sparse having very few (2–3) weeks of reported cases that produced very high (≥ 9) and likely spurious *R*_*0*_ values [18]. Data from Puerto Rico were not used because case numbers reported in the second and subsequent weeks of reporting (mean 428 per week) were stable and an order of magnitude lower than the number of cases reported in the first week (2705). These data also resulted in a very high estimated *R*_*0*_ value, which we considered likely spurious due to some anomaly in the reporting of cases at the time of data extraction. Consequently, for analysis there were data from 24 countries for which estimates of *R*_0_ could be obtained, and for which there were ZIKV test result data for Canadian travellers. The latter comprised 3551 test results, of which 92 (2.59%, exact 95% confidence interval [95% CI] = 2.09–3.17%) were positive. The prevalence of positive test results amongst countries varied from 0 to 33.3% (Fig. 1).

### Estimation of *R*_*0*_

**Fig. 2:**
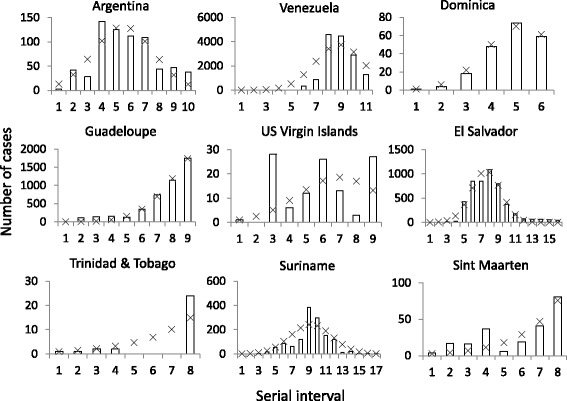
Example surveillance data from ZIKV-affected countries. The *bars* show the reported numbers of cases (suspected and confirmed cases combined) by 16 day serial interval period. The *crosses* show the predicted number of cases using the parameters obtained by the IDEA model [18]

**Fig. 3:**
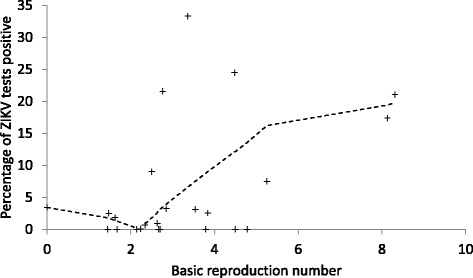
The relationship of estimates of *R*_*0*_ for countries in Central and South America and the Caribbean, to the prevalence of positive ZIKV test results for travellers to those countries. *Crosses* indicate prevalence values in travellers to an individual country and the *dashed line* is lowess smoothed estimates of prevalence. The identified cut-off *R*_*0*_ value that dichotomised countries into high-risk and low-risk for travellers was 2.76, and there were 12 countries in each of these groups

Case numbers predicted by the parameter estimates of the IDEA model were in most cases consistent with observed case numbers (Fig. 2). Estimates of *R*_*0*_ values obtained for the 24 countries ranged from approaching zero to 8.31 (Fig. 1). The prevalence of positive ZIKV test results varied non-linearly with *R*_*0*_ estimates for the countries (Fig. 3).

### Receiver operating characteristic analysis

The ROC AUC value for estimates of *R*_*0*_ as predictors of the ZIKV test result was good (AUC = 0.83, standard error = 0.02, 95% CI = 0.79–0.87) according to accepted criteria [26]. A cut-off value of ≥ 2.76 for *R*_*0*_ was chosen to dichotomise the ZIKV test data, which gave the greatest combined specificity and sensitivity (73.6 and 84.6%, respectively).

### Logistic regression analysis

In logistic regression with country ID included as a random effect, travellers to countries with an estimated *R*_*0*_ ≥ 2.76 were 11 times more likely to test positive than travellers to countries with an estimated *R*_*0*_ < 2.76 (odds ratio = 11.13; 95% CI = 3.14–39.25; Wald *Z* = 3.73, *P* < 0.001).

The proportions of travellers testing positive after travelling to Zika-affected countries with an estimated *R*_*0*_ ≥ 2.76 and < 2.76 were 11.2% (67/596) and 0.85% (25/2955), respectively.

### Sensitivity analysis

**Fig. 4:**
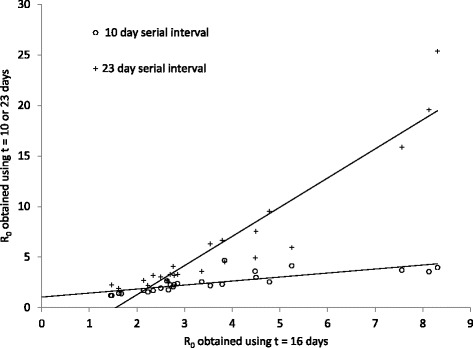
The relationship between *R*_*0*_ estimated using *t* = 10 (*circles*) or *t* = 23 days (*crosses*), and *R*_*0*_ estimated using *t* = 16 days

The *R*_*0*_ values obtained with 10 and 23 day values for *t* were respectively lower and higher than the values obtained using *t* = 16 days in most cases (Fig. 4). However, for Saint Lucia and Curacao, *R*_*0*_ values obtained with 10 and/or 23 day values for *t* could not be obtained due to the IDEA model not converging and data from these countries were not used in analyses.

ROC AUC values for the remaining data were the same when *t* = 16 days and *t* = 23 days but slightly lower when *t* = 10 days (Table 1). However there was no significant difference between these AUC values (*χ* ^2^ = 1.06, *P* > 0.1). The optimal cut-off values for dichotomising the *R*_*0*_ values were ≥ 1.93 when *R*_*0*_ values were obtained with *t* = 10 days and ≥ 3.30 when *R*_*0*_ values were obtained with *t* = 23 days. When using these cut-off values the proportions of positive test results in high and low *R*_*0*_ groups were the same when *t* = 23 days, but different when *t* = 10 days (Table 2), which was reflected in the results of logistic regression (Table 3). When *t* was 23 days the countries falling in the high and low risk groups were the same as when *t* was 16 days. However when *t* was 10 days, Colombia, the Dominican Republic and Guatemala were classified as belonging to the high risk group, while they were classified in the low risk group when using *t* set at 16 and 23 days.

**Table 1:** Results of ROC analysis for *R*_*0*_ as a predictor of the ZIKV test results, when *R*_*0*_ was estimated using *t* = 10, 16 and 23 days. Note that data from Saint Lucia and Curacao were not available for the sensitivity analysis

*t*-value used to estimate *R*_*0*_	ROC AUC	SE	95% CI
10 days	0.77	1.27	0.79–0.87
16 days	0.79	2.34	0.74–0.84
23 days	0.79	2.34	0.74–0.83

*Abbreviations*: *ROC AUC* receiver-operating characteristic area under the curve, *SE* standard error, *CI* confidence interval

**Table 2:** The proportions of travellers positive in high and low risk countries determined using cut-off levels for *R*_*0*_ when *R*_*0*_ was estimated using *t* = 10, 16 and 23 days. Note that data from Saint Lucia and Curacao were not used to estimate proportions when *R*_*0*_ was estimated using *t* = 10 and 23 days

*t*-value used to estimate *R*_*0*_	Cut-off for *R*_*0*_ used to determine high and low risk countries	Proportion (%) positive in ‘high risk’ group	Proportion (%) positive in ‘low risk’ group
10 days	1.98	86/1480 (5.8)	5/2042 (0.2)
16 days	2.76	67/596 (11.2)	25/2955 (0.1)
23 days	3.30	67/596 (11.2)	24/2905 (0.1)

**Table 3:** Parameter estimates from logistic regression models in which the ZIKV test result was the outcome and risk group (based on cut-off levels for *R*_*0*_ when *R*_*0*_ was estimated using *t* = 10, 16 and 23 days) was the explanatory variable

*R*_*0*_ estimated using	Odds ratio	95% CI	Wald *z*	*P*
*t* = 10 days	21.76	4.39–108.85	3.77	< 0.001
*t* = 16 days	11.13	3.12–39.25	3.73	< 0.001
*t* = 23 days	11.71	3.09–44.70	3.61	< 0.001

*Abbreviation*: *CI* confidence interval

## Discussion

Estimates of *R*_*0*_ for ZIKV in outbreak-affected countries correlated with the proportions of travellers acquiring ZIKV. Therefore risk to travellers varies with the force of transmission cycles in the countries they are visiting, which suggests that travellers are not a group that is particularly highly protected from infection in affected countries by virtue of their traveller status. This raises the possibility that different levels of ZIKV infection risk in different countries may be identified and possibly communicated to travellers. The need for such nuanced risk communication will become increasingly important if ZIKV spreads more widely in the world, in which case simply not visiting affected countries will become a decreasingly viable decision for many travellers. Second, this study suggests that estimates of *R*_*0*_ (or other analyses of the force of infection) in affected countries could be used to enhance methods used to assess the capacity for ZIKV spread by travellers, which often account for within-source country incidence using simple indices [14, 29] or not at all [30]. Also, these estimates could provide useful validation data for models predicting importation and spread of ZIKV that are being developed [31]. Such models predict actual numbers of cases rather than the numbers reported in surveillance, which vary amongst countries according to surveillance systems and effort, as well as according to the dynamics of ZIKV transmission. The method we used here obtains an estimate of *R*_*0*_ based on the shape of the epidemic curve rather than the reported incidence so it allows comparisons to be made amongst jurisdictions in which surveillance effort may be different.

The relationship between estimated *R*_*0*_ for the ZIKV-affected countries and infection prevalence in Canadian travellers was nonlinear and we chose to use a simple *R*_*0*_ cut-off point to dichotomise the data for analysis. Why this relationship was a step function is not clear. One possible explanation is background noise within the reported ZIKV case numbers, associated with false positivity and negativity, which may mask the real status of transmission when incidence is low. More research and data from more countries and time points during epidemics are needed to better understand the relationship between *R*_*0*_ and incidence in travellers. Nevertheless, the difference in incidence in travellers exposed to countries above and below the identified *R*_*0*_ cut-off (2.76) was stark. The likelihood of testing positive was an order of magnitude greater in travellers to countries with *R*_*0*_ values at or above the cut-off compared to travellers to countries with *R*_*0*_ values below the cut-off. The estimates of *R*_*0*_ for each country provided an index of the status of Zika transmission and risk of infection for travellers despite variations amongst countries in surveillance effort and case definition, and the fact that we are estimating *R*_*0*_ mostly on the basis of symptomatic cases, when most ZIKV infections are asymptomatic [2]. For these reasons, the value of *R*_*0*_ = 2.76 may not be a hard and fast cut-off figure, ready to use for developing risk communications. It reflects the status of data we have to present at the onset of the outbreak, both on Canadian travellers and on surveillance in affected countries, and further study will be needed to confirm its appropriateness for development of sound risk communications.

We were not able to obtain an *R*_*0*_ value for Brazil for the study period even though it is well recognised that Brazil suffered a very significant epidemic. The prevalence of ZIKV-positive test results in Canadian travellers (2%, 4/203 tests) was relatively low and more consistent with Brazil being in the low risk group in our study. This suggests that either Brazil was an outlier in terms of environmental risk for travellers equating with incidence of infection, or that the epidemic was largely over by the time many of the travellers visited Brazil. There is indeed some evidence that the latter was the true. Phylogeny-based estimates of the date of importation of ZIKV into Brazil are as early as 2013 [32], while evidence from surveillance for late-pregnancy microcephaly suggests that the epidemic peaked early in 2015 [33].

The *R*_*0*_ estimates were sensitive to the value for the serial interval, but identification of high and low risk countries using *R*_*0*_ estimates was robust to variations in the serial interval from 16 to 23 days. A serial interval of 10 days gave a qualitatively similar but quantitatively somewhat different classification of the countries. Ten days may, however, be biologically implausible as a mean serial interval, so *R*_*0*_ values generated using this interval could be expected to be less well predictive of the risk of traveller infection than when using more plausible 16 or 23 day serial intervals. The serial interval comprises the intrinsic incubation period in humans (minimally 3 days [19], but more likely 5 days and longer [34]), the extrinsic incubation period in mosquitoes (as short as 5 days experimentally [35] but in nature more likely ≥ 10 days [36]), the time mosquitoes take to feed twice, and considerations of how the gonotrophic cycle in the mosquitoes affects how frequently they feed [37]. Serial intervals have most frequently been estimated at ≥ 14 days for related flaviviruses [38, 39] so it would perhaps be unsurprising if the serial interval of ZIKV infections was similar.

There are other methods of estimating *R*_*0*_ or the effective reproduction number *R* from surveillance data (e.g. [40, 41] and articles reviewed in [12]), but the IDEA model approach was convenient because it provided an estimate of *R*_*0*_ while accounting for factors (including control, seasonal declines in vector numbers or herd immunity) that contribute to post-epidemic peak declines in incidence. Furthermore, it obtains estimates of *R*_*0*_ that allow comparisons to be made amongst jurisdictions in which surveillance effort is different. However, as time goes on, incidence will fall in affected countries for a range of reasons (e.g. development of herd immunity and control efforts), and ZIKV transmission will likely transition from epidemic to endemic status. Once this occurs, methods other than the IDEA model may be more appropriate for assessing risk to travellers from surveillance data.

## Conclusions

This study demonstrated that risk to travellers in ZIKV-affected countries correlates with estimates of *R*_*0*_ for those countries obtained using human case surveillance data. This relationship was non-linear with risk of travellers testing positive being over tenfold greater in countries with an estimated *R*_*0*_ equal to or greater than 2.76. This suggests that estimates of the force of infection in ZIKV-affected countries may be use to nuance risk information for travellers, and help predict rates of spread of ZIKV from those countries.

## Additional files

Additional file 1: ZIKV infection surveillance case definitions. (DOCX 57 kb)
Additional file 2: Data used in this study. (XLSX 13 kb)

## Abbreviations

95% CI: 95% confidence interval
AUC: Area under the curve
CDC: United States Centers for Disease Control and Prevention
IDEA: Incidence decay and exponential adjustment model
MAC-ELISA: IgM-antigen capture enzyme-linked immunosorbent assay
NML: National Microbiology Laboratory
PAHO: Pan American Health Organization
PRNT: Plaque reduction neutralization test
*R*_*0*_: The basic reproduction number
ROC: Receiver operating characteristic
RT-PCR: Reverse transcriptase polymerase chain reaction
SE: Standard error
ZIKV: Zika virus
