# Supplementary material for: Risk of travel-related cases of Zika virus infection is predicted by transmission intensity in outbreak-affected countries
Source: Parasit Vectors. 2017 Jan 25;10:41. doi: 10.1186/s13071-017-1977-z (PMC5264286; doi:10.1186/s13071-017-1977-z)
Supplement: Additional file 1: — ZIKV infection surveillance case definitions. (DOCX 57 kb) [file 13071_2017_1977_MOESM1_ESM.docx]

**Section 1: Case definitions**

Table 1: Zika virus infection case definitions by country.

| **Country** | **Suspected Case Definition** | **Confirmed Case Definition** | **Source of Case Definitions** | **Surveillance Reports** |
| --- | --- | --- | --- | --- |
| Argentina | Unavailable | Unavailable | Argentina Ministry of Health (MOH) website:  <http://www.msal.gob.ar/> | Weekly Epidemiological bulletins:  <http://www.msal.gob.ar/index.php/home/boletin-integrado-de-vigilancia> |
| Aruba | Unavailable | Unavailable | Aruba MOH website:  <http://www.despa.gov.aw/> | Unavailable |
| Barbados | Unavailable | Unavailable | Barbados MOH website: <http://www.health.gov.bb/index.php/ministry_of_health> | Unavailable |
| Belize | Unavailable | Suspected samples are sent to *The Caribbean Public Health Agency (CARPHA)* for confirmatory testing by RT-PCR^*^ | Epidemiological alert:  <http://health.gov.bz/www/attachments/article/878/PAPU%20Newsletter%202016_Volume%202,%20Issue%201.pdf> | Press releases documenting new cases are available at:  <http://health.gov.bz/www/index.php> |
| Bolivia | Unavailable | Unavailable | Bolivia MOH website:  <https://www.minsalud.gob.bo/> | Unavailable |
| Bonaire | Unavailable | Unavailable | Netherlands MOH website:  <http://www.rivm.nl/en> | Unavailable |
| Brazil | Patients with a pruritic maculopapular rash and two or more of the following signs and symptoms:   - Fever - Conjunctival hyperemia without secretion and itching - Polyarthralgia - Periarticular edema | A suspected case with a positive diagnosis based on one of the following laboratory tests:   - Virus isolation - Detection of viral RNA by reverse transcriptase reaction (RT-PCR); - IgM serology   ( For laboratory algorithms see guidelines) | Surveillance Guidelines:  <http://portalsaude.saude.gov.br/images/pdf/2016/marco/07/Nota-Informativa-zika.pdf> | Epidemiological bulletins:  <http://combateaedes.saude.gov.br/en/epidemiological-situation> |
| Colombia | 3 different suspected case definitions have been developed for different subsets of the population. These definitions can be found on page 8 of the surveillance guidelines. | Different confirmed case definitions have been developed for different subsets of the population. These definitions can be found on page 8 of the surveillance guidelines. | Surveillance Guidelines:  <http://www.ins.gov.co/lineas-de-accion/Subdireccion-Vigilancia/sivigila/Protocolos%20SIVIGILA/PRO%20Zika.pdf> | Weekly Epidemiological bulletins:  <http://www.ins.gov.co/boletin-epidemiologico/Paginas/default.aspx> |
| Costa Rica | Patient presenting with rash (maculopapular and pruritic) and two or more of the following signs or symptoms:   - Fever, usually <38.5 ° C - Conjunctivitis (Nonpurulent / hyperemia) - Arthralgia - Myalgia - Edema periarticular   And that:   - In the 2 weeks prior to the onset of symptoms have a history of residence or travel to an area with local transmission of the virus or with presence of the vectors.  OR - Had sexual contact without protection in the 2 weeks prior to the onset of symptoms, with a person who in the 8 weeks prior to sexual contact has a history of residence or travel to an area with local transmission of Zika or with the presence of vectors. | Any suspected case where Zika is detected by RT-PCR | Surveillance Guidelines:  <https://www.ministeriodesalud.go.cr/index.php/vigilancia-de-la-salud/normas-protocolos-y-guias/2996-lineamientos-nacionales-para-la-enfermedad-por-virus-zika-actualizado-al-6-de-abril-2016/file> | Weekly Epidemiological bulletins:  <https://www.ministeriodesalud.go.cr/index.php/vigilancia-de-la-salud/analisis-de-situacion-de-salud> |
| Cuba | Unavailable | Unavailable | Cuba MOH website^:^  <http://www.sld.cu/> | Unavailable |
| Curacao | Unavailable | Unavailable | Netherlands MOH website:  <http://www.rivm.nl/en> | Unavailable |
| Dominica | Unavailable | Unavailable | Dominica MOH website:  <http://dominica.gov.dm/search-results2?searchword=zika&searchphrase=all> | Unavailable |
| Dominican Republic^^^ | Following PAHO’s exact case definition | Following PAHO’s exact case definition | Surveillance Guidelines:  <http://digepisalud.gob.do/docs/Vigilancia%20Epidemiologica/Alertas%20epidemiologica/Zika/Nacional/Procedimiento_Vigilancia_ZIKV_2016_Ene_31.pdf> | Weekly Epidemiological bulletins:  <http://digepisalud.gob.do/documentos/?drawer=Boletines%20epidemiol%C3%B3gicos*Boletines%20semanales*2016> |
| Ecuador | Unavailable | Unavailable | Surveillance Guidelines (case definitions not specified):  <http://www.salud.gob.ec/wp-content/uploads/2015/12/BOLETIN-NO.-4-ZIKA-1.pdf> | Epidemiological bulletins:  <http://www.salud.gob.ec/zika/> |
| El Salvador | Any patient with a fever >38.5º C for 2 or more days, a maculopapular rash, non-purulent conjunctivitis accompanied by edema of the limbs, arthritis or arthralgia. Furthermore, a person that has had contact with a confirmed case of Zika, or someone with similar symptoms within the last 15 days OR a person that has remained in a country with active Zika virus transmission 15 days before the onset of symptoms. | Any suspected case where Zika is detected by RT-PCR | Surveillance Guidelines <http://www.salud.gob.sv/archivos/vigi_epide2015/boletines_epidemilogicos2015/Boletin_epidemiologico_SE412015.pdf> | Weekly Epidemiological bulletin:  <http://www.salud.gob.sv/tag/boletines-epidemiologicos-2016/> |
| French Guiana | A patient who has been presenting for less than 7 days with the following symptoms:   - A maculopapular rash with or without fever; AND   At least two of the following signs: conjunctival hyperemia; arthralgia; myalgia; in the absence of other etiology’s. | A suspected case in which the viral genome Zika is detected in the blood or urine by RT-PCR | Surveillance Guidelines:  <http://www.invs.sante.fr/Dossiers-thematiques/Maladies-infectieuses/Maladies-a-transmission-vectorielle/Zika/Le-systeme-de-surveillance> | Reports for the overseas departments of France:  <http://www.invs.sante.fr/Dossiers-thematiques/Maladies-infectieuses/Maladies-a-transmission-vectorielle/Zika/Donnees-epidemiologiques> |
| Grenada | Unavailable | Unavailable | Grenada MOH website:  <http://www.health.gov.gd/index.php?lang=en> | Unavailable |
| Guadeloupe | A patient who has been presenting for less than 7 days with the following symptoms:   - A maculopapular rash with or without fever; AND   At least two of the following signs: conjunctival hyperemia; arthralgia; myalgia; in the absence of other etiology’s. | A suspected case in which the viral genome Zika is detected in the blood or urine by RT-PCR | Surveillance Guidelines:  <http://www.invs.sante.fr/Dossiers-thematiques/Maladies-infectieuses/Maladies-a-transmission-vectorielle/Zika/Le-systeme-de-surveillance> | Reports for the overseas departments of France:  <http://www.invs.sante.fr/Dossiers-thematiques/Maladies-infectieuses/Maladies-a-transmission-vectorielle/Zika/Donnees-epidemiologiques> |
| Guatemala | A patient presenting with a fever greater than or equal to 38.5^o^ C, with arthralgia and nonpurulent conjunctivitis with or without any of the following symptoms:   - Headache - Myalgia - Asthenia - Maculopapular rash - Edema of the lower limbs - Retro-orbital pain - Anorexia, vomiting, diarrhea, abdominal pain   Also, the patient has traveled or resides in an area with active Zika transmission | Any suspect or probable case with a positive laboratory test for Zika virus | Surveillance Guidelines:  [http://epidemiologia.mspas.gob.gt/files/Protocolo%20Zica.pdf](http://epidemiologia.mspas.gob.gt/files/Protocolo%20Zica.pdf%20) | Weekly Epidemiological bulletins:  <http://epidemiologia.mspas.gob.gt/files/Publicaciones%202016/Boletines/Boletin%20semana%20epidemiologia.pdf> |
| Guyana | Unavailable | Unavailable | Guyana MOH website:  <http://www.health.gov.gy/> | Unavailable |
| Haiti | Unavailable | Unavailable | Haiti MOH website:  <http://mspp.gouv.ht/newsite/> | One epidemiological has been posted:  <http://mspp.gouv.ht/site/downloads/Evolution%20epidemie%20du%20ZIKA%20en%20Haiti%20au%203%20fev%202016.pdf> |
| Honduras | Unavailable | Unavailable | Honduras MOH website  <http://www.salud.gob.hn/web/> | Unavailable |
| Jamaica | Patients with a rash or elevated body temperature (> 37.2 °C) and with one or more of the following symptoms (not explained by other medical conditions):   - Arthralgia or myalgia - Non-purulent conjunctivitis or conjunctival hyperaemia - Headache or malaise | A suspected case with a laboratory positive result for the specific detection of Zika virus | Surveillance Guidelines:  <http://moh.gov.jm/wp-content/uploads/2015/12/ZIKA-MOH-EPIDEMIOLOGICAL-SURVEILLANCE-PLAN-FINAL-NOV-16-2015.pdf> | Unavailable |
| Martinique | A patient who has been presenting with the following symptoms for less than 7 days:   - A maculopapular rash with or without fever; AND   At least two of the following signs: conjunctival hyperemia; arthralgia; myalgia; in the absence of other etiology’s. | A suspected case in which the viral genome Zika is detected in the blood or urine by RT-PCR | Surveillance Guidelines:  <http://www.invs.sante.fr/Dossiers-thematiques/Maladies-infectieuses/Maladies-a-transmission-vectorielle/Zika/Le-systeme-de-surveillance> | Epidemiological reports for the overseas departments of France:  <http://www.invs.sante.fr/Dossiers-thematiques/Maladies-infectieuses/Maladies-a-transmission-vectorielle/Zika/Donnees-epidemiologiques> |
| Mexico | Patient presenting with a rash (usually maculopapular and pruriginous) and two or more of the following signs or symptoms :   - - Fever   - Headache   - Conjunctivitis   - Arthralgia   - Myalgia   - Periarticulr edema  PruritusRetroocular painAnd to identify any epidemiological associationPresence of the vector *Ades Aegypti* or *Aedes Albopictus* ORHistory of visiting or living in areas of transmission two weeks prior to the start of symptoms ORExistence of confirmed cases in the patients region ORHistory of sexual contact without protection in the two weeks prior to the appearance of symptoms, with a person who 8 weeks prior to sexual contact has a history of residence or travel to an area with local transmission of Zika or with presence of vectors | All probable cases with a positive result for Zika through the detection of viral RNA by RT-PCR in serum samples taken within the first five days of symptom onset | Surveillance Guidelines:  <http://www.epidemiologia.salud.gob.mx/doctos/lineamientos/2016/lineamientos_ve_y_lab_virus_zika.pdf> | Weekly Epidemiological bulletins:  <http://www.epidemiologia.salud.gob.mx/dgae/avisos/zika.html> |
| Nicaragua | Unavailable | Unavailable | Nicaragua MOH website:  <http://www.minsa.gob.ni/> | Press releases documenting positive laboratory tests are available on the MOH website. |
| Panama | Unavailable | Unavailable | Panama MOH website:  <http://www.minsa.gob.pa/> | Weekly Epidemiological bulletins:  <http://www.minsa.gob.pa/epidemiologia/zika-2016> |
| Paraguay^^^ | Following PAHO’s exact case definition | Following PAHO’s exact case definition | Surveillance Guidelines:  <http://vigisalud.gov.py/wp-content/uploads/2016/05/Vigilancia-Zika-Paraguay-2016-1.pdf> | Weekly Epidemiological bulletins:  <http://vigisalud.gov.py/index.php/boletin-epidemiologico/> |
| Peru | Unavailable | Unavailable | Peru MOH website: <http://www.dge.gob.pe/portal/> | Weekly Epidemiological bulletins:  <http://www.dge.gob.pe/portal/index.php?option=com_content&view=article&id=347&Itemid=249> |
| Puerto Rico | Unavailable | Confirmed cases include patients that meet the criteria for a confirmed case and/or a probable case as defined by the Council of State and Territorial Epidemiologists (CSTE). | Case definitions are documented in the weekly epidemiological bulletins  CSTE Guidelines:  <https://www.cste2.org/docs/Zika_Virus_Disease_and_Congenital_Zika_Virus_Infection_Interim.pdf> | Weekly Epidemiological bulletins:  <http://www.salud.gov.pr/Estadisticas-Registros-y-Publicaciones/Pages/Informe-Arboviral.aspx> |
| Saint Barthelemy | A patient who has been presenting for less than 7 days with the following symptoms:   - A maculopapular rash with or without fever; AND   At least two of the following signs: conjunctival hyperemia; arthralgia; myalgia; in the absence of other etiology’s. | A suspected case in which the viral genome Zika is detected in the blood or urine by RT-PCR | Surveillance Guidelines:  <http://www.invs.sante.fr/Dossiers-thematiques/Maladies-infectieuses/Maladies-a-transmission-vectorielle/Zika/Le-systeme-de-surveillance> | Epidemiological reports for the overseas departments of France:  <http://www.invs.sante.fr/Dossiers-thematiques/Maladies-infectieuses/Maladies-a-transmission-vectorielle/Zika/Donnees-epidemiologiques> |
| Saint Lucia | Unavailable | Unavailable | Saint. Lucia Zika Virus alert (case definitions not included):  <http://health.govt.lc/zika> | Unavailable |
| Saint Martin | A patient who has been presenting for less than 7 days with the following symptoms:   - A maculopapular rash with or without fever; AND  At least two of the following signs: conjunctival hyperemia; arthralgia; myalgia; in the absence of other etiology’s. | A suspected case in which the viral genome Zika is detected in the blood or urine by RT-PCR | Surveillance Guidelines:  <http://www.invs.sante.fr/Dossiers-thematiques/Maladies-infectieuses/Maladies-a-transmission-vectorielle/Zika/Le-systeme-de-surveillance> | Epidemiological reports for the overseas departments of France:  <http://www.invs.sante.fr/Dossiers-thematiques/Maladies-infectieuses/Maladies-a-transmission-vectorielle/Zika/Donnees-epidemiologiques> |
| Saint Vincent and the Grenadines | Unavailable | Suspected samples are sent to *The Caribbean Public Health Agency (CARPHA)* for confirmatory testing by RT-PCR^*^ | Article stating CARPHA is responsible for confirmatory testing:  <http://health.gov.vc/health/index.php?option=com_content&view=article&id=589:ministry-of-health-wellness-and-the-environment-advises-of-twenty-new-confirmed-cases-of-zika-virus-infection&catid=38:latest-news&Itemid=65> | Press releases documenting new confirmed cases are released at:  <http://health.gov.vc/health/index.php?option=com_content&view=category&id=38&Itemid=65> |
| Sint Maarten | Patient with rash or elevation of body temperature (> 37.2 °C) with one or more of the following symptoms (not explained by other medical conditions:   - Arthralgia or myalgia - Non-purulent conjunctivitis or conjunctival hyperemia - Headache or malaise | Any suspected case with a positive laboratory test for Zika virus (see surveillance guidelines for specific laboratory algorithm) | Surveillance Guidelines:  <http://www.sintmaartengov.org/government/VSA/Health-Updates/Documents/Zika%20Alert%20%20community.pdf> | Unavailable |
| Suriname | Unavailable | Unavailable | Suriname MOH website  [http://www.gov.sr/ministerie-van-volksgezondheid.aspx](http://www.gov.sr/ministerie-van-volksgezondheid.aspx%20) | Unavailable |
| Trinidad and Tobago | Unavailable | Suspected samples are sent to *The Caribbean Public Health Agency (CARPHA)* for confirmatory testing by RT-PCR^*^ | Article stating CARPHA is responsible for confirmatory testing:  http://www.news.gov.tt/features-and-analysis/health#.V4UId_kwhaR | Press releases documenting confirmed cases are released at:  http://www.news.gov.tt/features-and-analysis/health#.V4T2x9JIiRs |
| United States Virgin Island | Any resident or visitor to the USVI diagnosed by a health care provider with suspected Zika; regardless of subsequent laboratory testing. | A laboratory positive reported case with Zika virus- specific nucleic acid and in serum and/or IgM antibodies in serum and confirmatory plaque reduction neutralization testing to differentiate potential cross-reactive antibodies. | Case definitions are documented in the weekly epidemiological reports | Weekly epidemiological reports:  <http://www.healthvi.org/topics/az/z/zika.html> |
| Venezuela | Patient with rash or elevated body temperature (axillary ) > 37.2 °C and one or more of the following symptoms (which are not explained by other medical conditions):   - - Arthralgia or myalgia   - No purulent conjunctivitis or conjunctival hyperemia   - Headache or malaise | Any suspected case with a positive laboratory test for Zika virus (see surveillance guidelines for specific laboratory algorithm) | Surveillance Guidelines: <https://drive.google.com/file/d/0By6RZhEqt4ajY1RmU041b250WjQ/view?pref=2&pli=1> | Unavailable |

^*^ Zika Virus testing protocol for CARPHA is available at: <http://www.onecaribbean.org/wp-content/uploads/ZikaVirusSurveillance.pdf>
Information for this table was extracted up until July 7^th^, 2016

**PAHO Case Definitions:**

Suspected case of Zika virus disease:

Patient with rash* with **two or more** of the following signs or symptoms:

- fever, usually <38.5 ° C
- conjunctivitis (non-purulent/hyperemic)
- arthralgia
- myalgia
- peri-articular edema

*usually pruritic and maculopapular

Suspected case of Zika virus disease in geographic areas without autochthonous transmission and where there are no vectors present:

Patient who meets the criteria for a suspected case (above) **AND** who

- in the 2 weeks prior to onset, traveled to, or resided in, a geographic area where there is known local transmission of the Zika virus or there is known vector presence;  **OR**
- had un-protected sex, in the 2 weeks prior to onset, with a person who traveled, in the previous 8 weeks, to a geographic area with (a) known local transmission of the Zika virus or (b) and area with known vector presence.

Confirmed case of Zika virus disease:

Patient who meets the criteria for a suspected case **AND** has laboratory confirmation of recent Zika virus infection, i.e.:

- RNA or Zika virus antigen in any specimen (serum, urine, saliva, tissue or whole blood);  **OR**
- Positive Zika IgM antibodies AND Plaque reduction neutralization (PRNT90) for Zika virus titers = 20 and four or more times greater than the titers for other flaviviruses; AND exclusion of other flavivirus; **OR**
- In autopsy specimens, detection of the viral genome (in fresh or paraffin tissue) by molecular techniques, or detection by immuno-histochemistry.

Pan American Health Organization (2016). Case definitions. Available at: http://www.paho.org/hq/index.php?option=com_content&view=article&id=11117&Itemid=41532&lang=en

Out of the 39 countries for which PAHO has reported data, 18 have not publicly released Zika virus case definitions, 16 have both a confirmed and suspected case definition, and 5 only have a confirmed case definition. Only two countries, Paraguay and Dominican Republic, have explicitly stated that they are using PAHO’s case definitions. However, case definitions across countries are very similar, and have only slight variations from the case definition developed by PAHO.
